# Complement Dependent Synaptic Reorganisation During Critical Periods of Brain Development and Risk for Psychiatric Disorder

**DOI:** 10.3389/fnins.2022.840266

**Published:** 2022-05-06

**Authors:** Laura J. Westacott, Lawrence S. Wilkinson

**Affiliations:** ^1^Neuroscience and Mental Health Innovation Institute, MRC Centre for Neuropsychiatric Genetic and Genomics, School of Medicine, Cardiff University, Cardiff, United Kingdom; ^2^Hodge Centre for Neuropsychiatric Immunology, School of Medicine, Cardiff University, Cardiff, United Kingdom; ^3^Behavioural Genetics Group, Schools of Psychology and Medicine, Cardiff University, Cardiff, United Kingdom

**Keywords:** immune system, complement system, synaptic pruning, neurodevelopment, psychiatric disorder, adolescence, sensitive (critical) periods

## Abstract

We now know that the immune system plays a major role in the complex processes underlying brain development throughout the lifespan, carrying out a number of important homeostatic functions under physiological conditions in the absence of pathological inflammation or infection. In particular, complement-mediated synaptic pruning during critical periods of early life may play a key role in shaping brain development and subsequent risk for psychopathology, including neurodevelopmental disorders such as schizophrenia and autism spectrum disorders. However, these disorders vary greatly in their onset, disease course, and prevalence amongst sexes suggesting complex interactions between the immune system, sex and the unique developmental trajectories of circuitries underlying different brain functions which are yet to be fully understood. Perturbations of homeostatic neuroimmune interactions during different critical periods in which regional circuits mature may have a plethora of long-term consequences for psychiatric phenotypes, but at present there is a gap in our understanding of how these mechanisms may impact on the structural and functional changes occurring in the brain at different developmental stages. In this article we will consider the latest developments in the field of complement mediated synaptic pruning where our understanding is beginning to move beyond the visual system where this process was first described, to brain areas and developmental periods of potential relevance to psychiatric disorders.

## Introduction

The complement system is a key component of the immune system that plays a crucial role in inflammation and host-defence, yet in the central nervous system evidence is emerging that complement has important functions in homeostatic brain processes, beyond its canonical immune roles (Coulthard et al., 2018a; Hammad et al., 2018). In particular, a large body of evidence points to the involvement of complement proteins in developmental activity-dependent synaptic pruning (Stevens et al., 2007). There is also substantial and increasing evidence that complement is causally involved in the pathogenesis of neurodevelopmental and other psychiatric conditions. Alterations in peripheral complement protein levels and activation have been described in several disorders, including autism-spectrum disorder (ASD) (Corbett et al., 2007) schizophrenia (Maes et al., 1997), major depressive disorder (Ruland et al., 2016) bipolar disorder (Song et al., 2015) and post-traumatic stress disorder (Oganesyan et al., 2009). In the case of schizophrenia, in 2017 a landmark study uncovered that structural variation in the complement *C4A* locus is associated with risk of developing the disease (Sekar et al., 2016). This variant remains the strongest polygenic risk factor for schizophrenia identified to date. Since increasing *C4A* copy number predicts greater serum C4 levels, these pathogenic variants are likely to increase complement activation. Given the ability for activated complement molecules to tag synapses for elimination, excessive complement-mediated synaptic pruning is a highly plausible biological mechanism accounting for long-standing observations of grey matter reduction and synapse loss in schizophrenia (Feinberg, 1982). Consistent with this prediction, subsequent *in vitro* work demonstrated increased microglial engulfment of synaptic material in cells isolated from patients carrying schizophrenia risk associated variants (Sellgren et al., 2019) and that human *C4A* is more efficient at tagging synapses than the *C4B* isoform which is not associated with schizophrenia risk (Yilmaz et al., 2021). It should be noted that although the *C4A* alleles identified strongly associate with schizophrenia risk (Sekar et al., 2016), being a polygenic disorder these structural variants are likely to be present only in a subset of individuals with schizophrenia. Therefore, complement variants may contribute to synaptic deficits in a subset of people with the disorder, likely in interaction with other factors such as environmental risk. Nonetheless, given that schizophrenia genetic risk variants likely interact and converge on common biological pathways (Hall et al., 2015; Kim et al., 2021) studying the effects of the C4A variant will give insight into pathology relevant to schizophrenia as a whole.

On the other hand, in ASD a greater prevalence of the *C4B* gene null allele is found (Mostafa and Shehab, 2010) and C4B protein levels are decreased accordingly (Warren et al., 1995) although no associations between ASD and complement have been found at genome-wide significance level to date (Grove et al., 2019). In contrast to schizophrenia, ASD is characterised by increased dendritic spine density (Hutsler and Zhang, 2010; Weir et al., 2018) possibly due to an underactive synapse elimination processes early in life. In addition, greater amygdalar, hippocampal and cortical volumes have been reported in ASD (Schumann et al., 2004; Hutsler and Zhang, 2010) and these regions display altered developmental trajectories compared to typical development (Schumann et al., 2004). There is also evidence that the complement system can impact on fear and anxiety behaviour (Westacott et al., 2022) as well as cognition (Westacott et al., 2021) and mood (Crider et al., 2018), psychological domains frequently perturbed in psychiatric disorders. This raises the fundamental question of whether complement-dependent synaptic pruning in brain areas subserving these functions may contribute to clinical presentation and symptomatology in psychosis and other psychiatric disorders. However, there remains a significant gap in our understanding in the context of psychopathology as currently much of the literature on complement-dependent synaptic pruning has focused on early maturing brain regions such as the visual system, due in part to their ease of access and clearly defined periods of pruning. As such, there remain challenges in understanding how complement-mediated pruning mechanisms might apply to the rest of the brain, as well as other periods of development, such as adolescence when notably there is an increased risk for development of psychiatric disorder.

## Windows of Brain Maturation and Synaptic Reorganisation

A key feature of neurodevelopment is the occurrence of sensitive or critical periods during the lifespan, characterised by heightened sensitivity of the brain to environmental stimuli that influence developmental programs leading to normal brain function (Rice and Barone, 2000). Normal development is a consequence of a complex program of events including cell proliferation and migration, differentiation, network formation, maturation and refinement. These sequences of events are guided by endogenous cues as well as exogenous systemic or environmental cues. During particular epochs, exposure to particular stimuli is ‘anticipated’ and required to induce the plasticity and circuit refinement necessary to develop or shape given functions (Fuhrmann et al., 2015). An inevitable consequence of this is that the nervous system is increasingly vulnerable to insults that may disrupt the normal ontogeny of developmental processes (Rice and Barone, 2000). Of note, sensitive or critical periods can be distinguished by their ultimate impact on brain structure and function. Sensitive periods are thought to reflect gradual stages over development where certain functions or circuits are more malleable by experience, whereas critical periods are discrete periods of time in which experience is necessary to instigate more fundamental changes in neural networks (Rice and Barone, 2000; Knudsen, 2004; Fuhrmann et al., 2015; Nathalie Dehorter, 2020). Such critical periods have been discovered across different sensory modalities, but the most thoroughly characterised is in the visual cortex where the establishment of ocular dominance takes place during a brief postnatal window (Wiesel and Hubel, 1963; Hensch, 2005). It is within this critical period of development that we have the most evidence for complement playing a vital role in synaptic remodelling (Stevens et al., 2007; Schafer et al., 2012; Sekar et al., 2016).

Much research has focused on critical or sensitive periods occurring in childhood, but it has been proposed that adolescence constitutes a second ‘window of opportunity’ in brain development (Fuhrmann et al., 2015). This transitional phase of life, which can variably encompass 9–18 years of age (Semple et al., 2013), starts with the onset of puberty, and while its end is not clearly delineable, it can be considered as the transition to independence or onset of adult behaviour (Child AHCP, 2003). Several aspects of behaviour and cognition show heightened sensitivity and plasticity in adolescence, including memory performance, risk-taking and the effects of drugs of abuse, as well as social stress (reviewed in Fuhrmann et al., 2015). Moreover, adolescence is associated with heightened risk for the development of several social-emotional psychiatric disorders (Rapee et al., 2019), and disorders such as schizophrenia, anxiety and depression often onset during late adolescence and early adulthood (Woodward and Fergusson, 2001; Paus et al., 2008; Gogtay et al., 2011). These conditions are often chronic in nature and associated with poorer later-life outcomes (Dunn and Goodyer, 2006; Bandelow and Michaelis, 2015), highlighting the need for an improved understanding of the underlying neurobiological mechanisms.

The enhanced vulnerability for psychiatric risk around adolescence is associated with changes in brain structure and function (Tamnes et al., 2013) including decreasing grey matter volumes, increasing white matter volumes (Giedd et al., 1999; Aubert-Broche et al., 2010; Pfefferbaum et al., 2013) and dendritic and synaptic reorganisation. In mammalian species, there is an overproduction of neurons, axons and their synapses during the early postnatal years, a subset of which are subsequently removed *via* mechanisms such activity-dependent pruning (Tau and Peterson, 2019; Faust et al., 2021). This process ensures redundant synapses are eliminated to promote efficiency and maturation of neural circuits. Generalised synaptic pruning begins early in infancy [and has been noted to coincide with the onset of ASD (Eltokhi et al., 2020)] but the peak of pruning is thought to occur during adolescence (Huttenlocher, 1979). Animal studies have shown that the onset of puberty triggers synaptic pruning (Koss et al., 2014; Drzewiecki et al., 2016; Afroz et al., 2017), indexed by decreases synapse or dendritic spine density. In agreement, early studies of post-mortem human brain samples from across the lifespan (Huttenlocher, 1979; Huttenlocher and de Courten, 1987; Huttenlocher and Dabholkar, 1997) and the equivalent in non-human primates (Bourgeois and Rakic, 1993) pointed to a decline in synaptic density in several cortical areas beginning in late childhood and culminating at the end of adolescence. Later work by Petanjek et al. (2011) in the human prefrontal cortex supported these initial studies but indicated that the elimination of synaptic spines continues beyond adolescence and into the third decade of life (Petanjek et al., 2011), evidencing an extremely protracted period of synaptic reorganisation, consistent with the prefrontal cortex being one of the last brain regions to mature (Gogtay et al., 2004) and potentially most vulnerable to dysfunction (Goto et al., 2010).

Despite these detailed studies of synaptic and spine density, for many years, precisely how such excess synapses were disposed of remained unknown. Over the last two decades however, a mounting body of evidence has established that proteins and cells canonically involved in the immune system participate in the removal of synapses during critical periods of development. Deficits in synaptic pruning, manifest as a persistence of immature synapses and connectivity beyond early critical periods, have been observed across multiple genetic models lacking proteins from the classical complement pathway [C1q, C3 (Stevens et al., 2007) and C4 (Sekar et al., 2016)] and microglial-signalling factors [e.g., complement receptor 3 or *CR3*, also known as *CD11b* (Schafer et al., 2012); the fractalkine receptor *Cx3Cr1* (Paolicelli et al., 2011; Zhan et al., 2014); and the microglia innate immune receptor *TREM2* (Filipello et al., 2018)]. While evident that multiple mechanisms for synaptic elimination exist, the complement system will be the focus of this review.

## Overview of the Complement System

**FIGURE 1:**
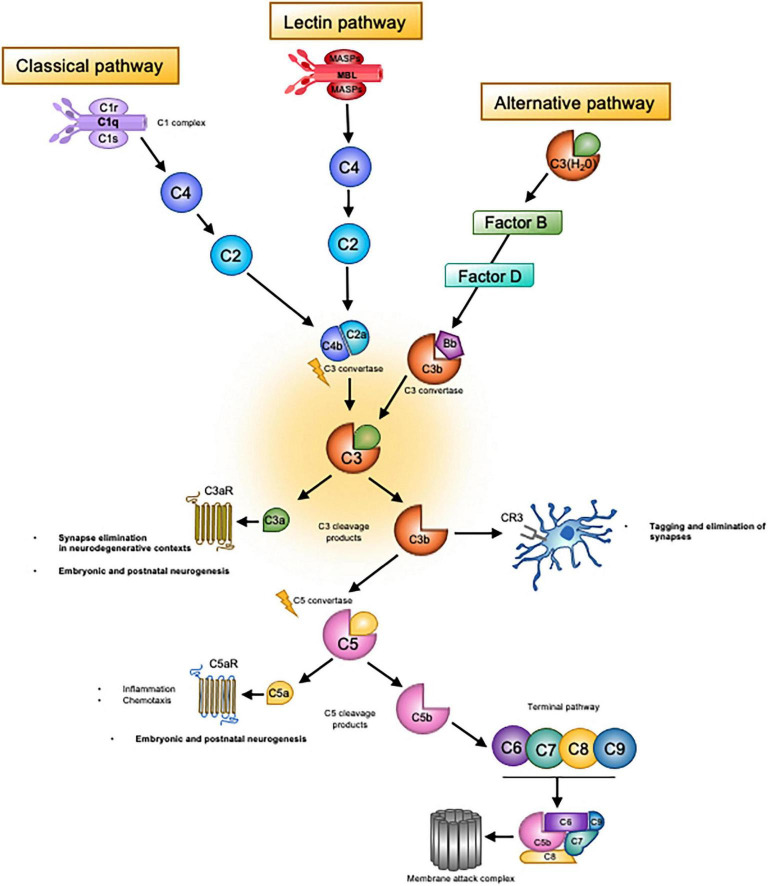
Overview of the complement system. Complement system consists of three main ‘recognition systems’; the classical pathway, the alternative pathway, the lectin pathway. Each pathway is initiated by different stimuli, which can be either exogenous or endogenous danger signals or pathogen-associated molecular patterns. Activation of the classical pathway is triggered by detection of antibody-antigen complexes by the initiator molecule of the classical pathway, C1q. C1q is then able to activate C1r and C1s, which cleave C4 and C2 to form the C3 convertase, C4b2a. The lectin pathway is initiated by the recognition of carbohydrates such as mannose upon pathogen surfaces by mannan-binding lectin (MBL), a molecule homologous to C1q of the classical pathway. MBL activates two serine proteases, MASP-1 and MASP-2, which then act to cleave classical pathway components C4 and C2. Activation then proceeds in the same manner as the classical pathway, eventually leading to generation of the C4b2a convertase. Unlike the classical and lectin pathways, the alternative pathway does not require pathogen recognition to initiate activation. Rather, there exists continuous low-level activation termed C3 tickover. In this process, the internal thioester bond of circulating C3 molecules is hydrolysed due to nucleophilic attack by H_2_O. This process occurs at a slow yet constant rate, leading to the formation of C_3_(H_2_O). After a series of interactions with C3b and regulatory molecules (Factor B and Factor D), the alternative pathway convertase is formed, C3bBb. Convertase complexes resulting from these distinct pathways then cleave C3, generating the main effectors of the complement system; C3b and C3a. The latter molecule, which is a small peptide, binds to its canonical receptor, the GPCR C3aR. The C3a/C3aR axis is a potent mediator of inflammatory and anti-inflammatory responses as well as a chemoattractant for macrophages and phagocytic cells. This axis appears to contribute to synaptic pruning in neurodegenerative contexts. As for C3b, upon deposition on surfaces of pathogens or host cells, this molecule attracts phagocytic macrophages, a process known as opsonisation, *via* complement receptor 3 (CR3). In the healthy developing brain, the C3b/CR3 axis has been shown to tag weak synapses for elimination by microglia bearing CR3. Unbound C3b molecules can also associate with other complement molecules to form a C5 convertase complex. C5 is then cleaved in a similar manner to C3, thereby generating cleavage fragments C5a and C5b, the former of which is another small peptide anaphylatoxin and inflammatory mediator. The cascade of activation continues with C5b and ultimately leads to assembly of the terminal complement effector; the membrane attack complex (MAC). Aggregation of MAC molecules on a target cell or pathogen creates pores in the cell membrane, leading to death by osmotic cell lysis.

Complement is an ensemble of over 40 fluid-phase and membrane associated proteins, receptors and regulators that collectively mediate immunosurveillance, inflammatory responses and regulation of adaptive immunity (see Figure 1; Beek et al., 2003). Classically, complement is organised into three distinct molecular pathways (though different modes of activation have been discovered); the classical, lectin and alternative, which each serve to recognise different microorganism-associated molecular patterns and endogenous danger signals, known as damage-associated molecular patterns (Orsini et al., 2014). Complement proteins circulate in an inactive state and are activated upon a proteolytic reaction whereby the protein is cleaved into smaller, active peptide fragments. These peptides then cleave downstream complement proteins, thereby propagating the cascade of activation in a sequential manner (Veerhuis et al., 2011; Orsini et al., 2014) to instigate the main functions of complement in inflammation, engulfment of foreign bodies or debris, or destruction of pathogens or cells *via* cell lysis. Activation of each complement pathway leads to the formation of a C3 convertase enzyme, which cleaves the central molecule C3, generating the main effectors of the complement system. Of note, the C4 complement protein, associated with schizophrenia risk in the landmark genome-wide significance study by Sekar et al. (2016), sits upstream of C3 within the classical pathway and serves to propagate C3 activity *via* formation of the C3 convertase complex (Figure 1). Cleavage of C3 produces two fragments, the inflammatory anaphylatoxin C3a and the opsonin C3b. The smaller C3a fragment binds to its canonical receptor, the G-protein coupled C3a receptor (C3aR) to stimulate pro and anti-inflammatory responses (Zhou, 2012). C3b on the other hand is an opsonin which facilitates removal by phagocytic cells. C3b molecules effectively coat the surfaces of cells or pathogens on which they are deposited, to enhance their recognition by phagocytes. The breakdown product of C3b, known as iC3b, is recognised by the complement receptor 3 (CR3, also known as cd11b/CD18) which is expressed by microglia in the brain (Stephan et al., 2012). Unbound C3b molecules can also associate with other complement molecules to form a C5 convertase complex which propagates further complement activation. C5 is cleaved in a similar manner to C3, generating cleavage fragments C5a and C5b. This cascade of activation ultimately leads to assembly of the terminal complement effector; the membrane attack complex (MAC). Accumulation of MAC molecules on a target cell or pathogen creates a pore in the cell membrane, leading to death by osmotic cell lysis (Morley and Walport, 1999). Complement is tightly controlled by a large group of regulatory molecules, which finely tune complement activation at a multitude of stages to prevent runaway activation leading to self-damage and inflammation (Morley and Walport, 1999; Veerhuis et al., 2011).

## Complement Expression and Activation in the Brain

It is known that the full complement system is expressed within the CNS, and there exists a baseline level of most complement components within cerebrospinal fluid under physiological conditions (Gasque et al., 1995; Morgan and Gasque, 1996), albeit at low levels compared to those found in infection or injury (Woodruff et al., 2010). For many years such observations of CNS complement expression were considered indicative of inflammation and thus detrimental to brain health, but this view has now dramatically changed owing to studies describing constitutive, physiological expression of complement expression during early prenatal and postnatal development (Veerhuis et al., 2011; Coulthard and Woodruff, 2015). The cellular sources of complement include neurons (Nataf et al., 1999), astrocytes (Sayah et al., 2003; Lian et al., 2015), vascular endothelial cells (Schraufstatter et al., 2002; Propson et al., 2021) and neural precursor cells (Rahpeymai et al., 2006), though transcriptome data indicates that microglia and macrophages are the principal cell type to express complement proteins, regulators and receptors (Zhang et al., 2014; Quell et al., 2017). Microglia in particular show ubiquitous expression of the complement receptor 3 (CR3) and also the C3a receptor (C3aR) (Quell et al., 2017).

While we know the brain is capable of expressing the entire complement system, it is *a priori* unlikely that the developmental roles carried out by these proteins require the full armoury of complement protein expression. Instead, fragmented expression of specific components of the complement system are found in a developmentally regulated and localised manner, suggesting dedicated functions of select pathways. For example, some complement proteins are present from the earliest stages of embryonic development. In xenopus embryos, C1qR, C3, C5 and C3aR expression is restricted to neural structures in the absence of terminal factors during neurulation (Mclin et al., 2002). The observation of localised expression of complement C1q, C3 and C4 proteins on the axons of retinal geniculate cells during highly specific periods of synaptic refinement (Stevens et al., 2007; Schafer et al., 2012) is consistent with this notion.

In addition, developmental actions of complement may not utilise the traditional mechanisms of activation seen in immune contexts (Figure 1). It is possible that the mechanism of C3-tickover, whereby the alternative pathway is constitutively activated at low levels to generate C3a and C3b (Lachmann, 2018), is responsible for complement activation under physiological conditions in the absence of pathogens or injury. In addition, other mechanisms through which complement can be activated have been discovered, including activation *via* a fourth ‘extrinsic’ pathway in which non-complement molecules such as thrombins mimic complement convertases to activate C3 and C5 (Huber-Lang et al., 2006). Furthermore, while it was long assumed that complement activation was restricted to the extracellular space, recent studies have revealed intracellular activation of complement, termed the ‘complosome’ (Arbore et al., 2017; Kunz and Kemper, 2021). Intracellular complement regulates metabolic re-programming of immune cells, and it has been speculated that this may contribute to known actions of T cells and cytokines such as IFN-γ and IL-10 on cognition and behaviour (Kemper and Köhl, 2018; Kunz and Kemper, 2021).

## Developmental Patterns of Complement Expression

Surprisingly, despite the association between complement, synaptic pruning and psychiatric disorders, there have been few investigations of complement expression levels during typical neurodevelopment. Evidence from preclinical model systems show that complement levels peak during early embryonic development (Mclin et al., 2002) with gradual decline during postnatal development and adult life, though individual complement proteins may present different patterns. In the mouse brain, the initiating complex of the classical pathway C1q increases dramatically in the ageing brain (Stephan et al., 2013; Fonseca et al., 2017) whereas C3 expression peaks in early postnatal development (Stevens et al., 2007), dropping by P30 with a subsequent increase in ageing (Reichwald et al., 2009; Shi et al., 2015; Propson et al., 2021) although other studies have reported increases as early as P60 in the healthy brain (Schwarz et al., 2012).

In humans, stable or increasing levels of serum complement proteins from infancy to adolescents and early adulthood have been reported (Willems et al., 2019). Since peripheral complement proteins are unlikely to cross the blood brain barrier, and the brain is capable of synthesising the entire complement system, systemic and central expression may be loosely associated at best. To our knowledge only one study has examined normal complement expression in the human brain from infancy to early adulthood. Sager et al. (2021) analysed complement gene expression in post-mortem dorsolateral prefrontal cortex (DLPFC) specimens from non-diseased human subjects (ranging from 2 months of age to 26 years). Elevated complement expression might be expected if complement-mediated synaptic pruning is a driving force behind adolescent synaptic refinement in this area, which is known to undergo protracted synaptic reorganisation lasting well into adulthood (Petanjek et al., 2011). However, C3, C1qb, C4 and CR3 showed peak expression between 1 and 5 years of age, with a subsequent decline of expression into adolescence (Sager et al., 2021). Moreover, complement regulatory proteins which inhibit activation were found to increase in expression from ages 5–12 and their expression was sustained through adolescence and early adulthood. Therefore, there does not seem to be a heightened expression of complement proteins in the healthy DLPFC during adolescence. Instead, the increased expression of complement control proteins suggests strong regulatory pressures on complement-mediated processes. Based on these data, the authors conclude that complement-mediated synaptic pruning may predominate earlier in development, between the ages of 1 and 5 instead of during adolescence (Sager et al., 2021). This may indicate that disrupted complement-mediated synaptic pruning would be of greater consequence in neurodevelopmental disorders that onset during this stage of life, such as ASD. Further studies will be required to examine the cellular source of complement proteins across development, and their correlation with synaptic densities. In addition, studies conducted in individuals with a diagnosis of schizophrenia or clinical high-risk groups will be important to determine whether dysregulation of these normal expression patterns during adolescence, for example as a consequence of the schizophrenia associated *C4A* genetic variant found to increase C4 copy number (Sekar et al., 2016) or variants in complement control genes (Håvik et al., 2011) contribute to pathogenesis of the disease.

## Sex Differences in Complement Activity

There is a significant gap in the literature pertaining to sex differences in complement expression patterns in the brain across development specifically. In human studies, biological males tend to show higher plasma levels of C3 and C4 than biological females, and these differences are most pronounced in individuals 20–50 years of age (Ritchie et al., 2004; Gaya da Costa et al., 2018; Kamitaki et al., 2020). Preclinical studies in rodents similarly demonstrate lower complement activity in females and resilience to complement driven pathologies, potentially a consequence of androgen regulation of complement production in the liver (Kotimaa et al., 2016). Interestingly, in animal studies, the onset of puberty triggered greater expression of innate immunity associated genes in males, and adaptive immunity related genes in females (Lamason et al., 2006) though complement gene expression specifically was not examined. Human data has described a potential sexual dimorphism in the expression of genes related to complement, innate immune activation and phagocytic processes (Prilutsky et al., 2017) whereby expression of this gene-set is enriched before birth in the male brain, but postnatally in the female brain. Sex differences in microglia are likely to be of relevance in understanding if and how complement expression varies between the sexes across development (VanRyzin et al., 2018). Transcriptome profiling of microglia has revealed sex-specific developmental gene expression patterns (Hanamsagar et al., 2017). In particular, areas including the amygdala and hippocampus harbour more microglia during early postnatal development in the male brain, whereas a greater abundance of activated microglia are found in the female brain between the onset of adolescence and early adulthood (Schwarz et al., 2012). Sex differences in microglia and complement likely interact with environmental risk factors to influence brain development and disease aetiology. In maternal immune activation (mIA) models, sex differences are seen in microglial expression of complement receptors and synaptic density (Hui et al., 2020) whereas prenatal exposure to toxic components of air pollution has been associated with altered microglial morphology, increased microglial-neuronal interactions and altered cortical volumes in male offspring only (Bolton et al., 2017). Further research on how biological sex influences complement and microglia interactions during development will be critical to understanding the pathophysiology of neurodevelopmental disorders which show sexually dimorphic prevalence rates such as ASD and schizophrenia.

## Mechanisms of Complement Mediated Synaptic Pruning

It is now well established that complement plays a critical role in the elimination of excess synapses during the narrowly defined window of development in which it has been most studied; the visual system. This has provided an excellent model system, since the segregation of eye-specific inputs that involves the pruning of overlapping axonal arbours of retinal ganglion cells is well understood and characterised, though previously little was known about the molecular mechanisms underlying synapse elimination (Stephan et al., 2012). In a series of studies, Stevens and colleagues used neuroanatomical tracing of retinogeniculate projections to show that mice deficient in C1q, C3, and CR3 have lasting deficits in eye-specific segregation, with dLGN neurons remaining binocularly innervated beyond the critical period of refinement (Stevens et al., 2007; Schafer et al., 2012). They posited that the opsonin iC3b tags weak or low-activity presynaptic inputs, thereby attracting microglia bearing CR3 which then phagocytose the synapse.

It is important to note that synapse elimination is not completely absent from C3 and C1q knockout mice (Stevens et al., 2007; Chu et al., 2010), however, demonstrating that complement is one of several mechanisms that control pruning. Other molecules such as the Class 1 major histocompatibility complex and pentraxins are also capable of driving pruning (Bjartmar et al., 2006; Boulanger, 2009; Lee et al., 2014). Complement-independent synaptic pruning has also been reported in regions including the barrel cortex (Gunner et al., 2019), and hippocampus (Paolicelli et al., 2011; Weinhard et al., 2018). Nonetheless, this work on complement-mediated synaptic pruning has been fundamental in transforming our understanding of the mechanisms underlying synaptic remodelling and brought about a new era of research focused on understanding how immune molecules are repurposed to support physiological processes in the brain. Despite this, there remain many unanswered questions, which we shall now consider in turn.

## Evidence for Complement-Mediated Pruning in Other Brain Regions Throughout the Lifespan

Human and animal studies have demonstrated that synapse density drops off in adolescence (Huttenlocher, 1979; Huttenlocher and de Courten, 1987; Petanjek et al., 2011) but to date most studies on complement-mediated synaptic pruning have focused on the first 1–3 postnatal weeks of rodent life, which is equivalent to approximately 23–40 weeks’ gestation in humans (Semple et al., 2013). While these studies have been extremely valuable in mapping out the foundational mechanisms underlying complement-mediated synaptic pruning, little is known of the role of complement in the synaptic pruning of later-maturing brain regions subserving cognitive, social and emotional functioning. Gaining an understanding of such areas are not without challenges however, since synaptic pruning is highly time and context dependent, dictated by the unique developmental trajectories of each region (Wierenga et al., 2014). In addition, such areas have considerably more complex circuitry than that of the visual retinogeniculate system and often pre- and post-synaptic partners are not clearly defined (Faust et al., 2021). Of note, there exists considerable variability in the definition of the adolescent period in rodents (Pinter et al., 2007). For the purposes of this review, we adopt the definition from Semple et al. (2013), of postnatal day (PND)35 to 49. This period in mice is roughly equivalent to 12–18 years of age in humans and includes the onset of puberty and eventual sexual maturity in both species.

An approximate time-course constructed from available evidence (including, but not restricted to complement-mediated synaptic pruning) is presented in Table 1 and demonstrates significant variation in the timing of pruning observed in different brain areas. Studies examining microglia-mediated synaptic pruning in mice have indicated that pruning peaks at PND 5 in the visual system (Stevens et al., 2007; Schafer et al., 2012; Sekar et al., 2016), between PND 15 to 4 months of age in the hippocampus (HPC) (Paolicelli et al., 2011; Zhan et al., 2014; Shi et al., 2015; Filipello et al., 2018), and not until PND 60 in the somatosensory cortex (Cong et al., 2020). In this section we will focus on the regions with the most evidence to date, that is the PFC and HPC.

**TABLE 1:** Estimated time course of microglia and/or complement dependent synaptic pruning in different brain regions.

Region	Study	Timepoints investigated (and metrics)	Peak of pruning or microglial activity identified?	Complement mediated?
DLGN	Stevens et al., 2007 Schafer et al., 2012 Sekar et al., 2016 Yilmaz et al., 2021	∼PND 10 (synapse/complement colocalisation, segregation of RGC projections) PND 5 (C3/VGLUT2 colocalisation, segregation of RGC projections) PND 0–10 (C3/VGLUT2 colocalisation, segregation of RGC projections) PND 0–10 (C4/VGLUT2 colocalisation, segregation of RGC projections)	All PND 5	Yes Yes Yes Yes
Hippocampus	Paolicelli et al., 2011 Filipello et al., 2018 Weinhard et al., 2018 Shi et al., 2015	PND 1-P40 (microglia density, dendritic spine density, PSD95 puncta) PND 18–20 (Iba1/CD68 volume, PSD95/VGLUT1 area, spine density, engulfed PSD95) PND 8–40 (Iba1/CD68 colocalisation) PND 30–16 months (C3/Homer colocalisation, synaptic puncta density)	P 15 N/a PND 15 4 months	Not investigated Not investigated N/a
PFC	Mallya et al., 2019 Yilmaz et al., 2021 Comer et al., 2020	PND 24–50 (spine density, microglia engulfment) PND 10–180 (synapse density, spine density, Iba1 CD68 volume) PND 7–60 (spine density, microglial engulfment)	PND 39 PND60 (humanised *C4A* mouse model) PND 21 (*C4A* overexpressing model)	Not investigated Yes Yes
Somatosensory cortex	Cong et al., 2020	PND 30–90 (C3 density, microglial engulfment, spine density, synaptic density)	PND60	Yes

Since human PFC maturation takes several years (Petanjek et al., 2011), synaptic refinement in the rodent PFC is likely to span a period of weeks. Peak microglial synapse engulfment has been observed at PND 39 in mice (Mallya et al., 2019), which coincides with the onset of puberty (Drzewiecki et al., 2016; Afroz et al., 2017) (puberty triggers the upregulation of innate immune genes in the periphery (Lamason et al., 2006), but it is not known whether this translates to increased complement activity). Two studies have investigated the impacts of the complement C4A variant associated with schizophrenia genetic risk on synaptic pruning in the PFC. Overexpression of human *C4A* lead to greater synapse engulfment from PND 10 to 40 in mice (Yilmaz et al., 2021). Dendritic spine density was reduced at PND 180, suggesting that excessive synaptic engulfment during this period lead to a loss of functional synapses. Another study of C4 overexpression in mice reported a reduction spine density in L2/3 of the PFC (Comer et al., 2020) where grey matter loss is most prominent in SCZ (Glantz and Lewis, 2000). This effect was observed at P21 suggesting complement mediated PFC synaptic pruning may commence pre-adolescence. These studies therefore implicate overexpression of C4 in excessive synaptic pruning beginning before pubescence and continuing throughout adolescence and provide a causal link between C4 variants identified in risk for schizophrenia and PFC dysfunction.

While C4 overexpression leads to overzealous pruning of PFC circuitry, C4 may not make a significant contribution to pruning of this region when expressed at physiological levels. Despite previous work showing synaptic elimination deficits in the visual system of C4 deficient mice during the first week of postnatal life (Sekar et al., 2016; see Table 1), Yilmaz et al. (2021) found no changes in synapse density in C4 knockout mice compared to wildtypes, suggesting that complement is not necessary for synaptic pruning to take place in this region. This seems to fit with observations that C4 protein is present at very low-levels in excitatory neurons of the PFC at P30 (Comer et al., 2020). In light of human PFC complement gene expression from the healthy brain, which does not support a role of complement in synaptic pruning at adolescence (Sager et al., 2021), it is possible that complement is otherwise redundant in the normal synaptic pruning processes taking place in the PFC during adolescence. Thus, perhaps is only in the context of overexpression (experimentally determined by C4A copy number) that C4 becomes a key player in aberrant synaptic pruning.

As for the hippocampus, microglial synaptic pruning (Paolicelli et al., 2011; Filipello et al., 2018) and/or partial phagocytosis [termed trogocytosis (Weinhard et al., 2018)] takes place prepubertally around PND 15 and is regulated by microglial signalling pathways (*Cx3cr1*, *TREM2*). With regard to complement singalling, one study found evidence of decreasing synaptic density between PND 30 and 4 months of age in wildtype mice, and a post-pubertal a peak in the colocalisation of the post synaptic density scaffold protein Homer and C3 was seen at 4 months of age suggesting prolonged adult pruning (Shi et al., 2015). A caveat of these data, however, is that no measures of microglial engulfment were reported. Nonetheless, the changes described were selective to CA3 but not CA1, although it was not associated with a loss of Homer^+^ puncta, but instead VGLUT2+ puncta were preferentially reduced. This is supported by other observations of VGLUT2+ synapse specific pruning (Salter et al., 2020) albeit this was observed at PND 16–18 in the CA1 region, suggesting the CA1 region may undergo pruning earlier than CA3. These differences in pruning may be linked to known distinctions in microglial reactivity in subregions of the hippocampus (Vinet et al., 2012). Nonetheless, in both studies, C3 deficient mice failed to show reductions in VGLUT2+ synapse density suggesting involvement of complement signalling in the elimination of these synapses (Shi et al., 2015; Salter et al., 2020). Together, these results suggest that there may be a prolonged second window of complement-mediated post-pubertal synaptic refinement in the hippocampus, in addition to an earlier pre-pubertal wave of pruning, and that complement may control the selective pruning of a subset of VGLUT2+ synapses.

Information regarding immune-mediated synaptic pruning in other brain regions is sparse. A priority area for future investigation is the amygdala, due to emerging links between aberrant synaptic pruning and anxiety. Abnormal anxiety is a frequent clinical feature of disorders such as schizophrenia and Alzheimer’s disease, for which genome-wide association studies pointing to the complement system highlight dysfunctional synaptic pruning as a candidate pathophysiological mechanism (Harold et al., 2009; Achim et al., 2011; Sekar et al., 2016; Hall, 2017). In addition, putative reductions in synaptic density indexed by synaptic vesicle glycoprotein 2A (SV2A) have been observed in the amygdala of anxious individuals post-mortem (Jurkiewicz et al., 2020). In the amygdala, connections between its basolateral and central nuclei control anxiety behaviours (Tye et al., 2011) and lesions of the ventral hippocampus reduce anxiety (Bannerman et al., 2003). These areas have protracted developmental trajectories extending well into adolescence (Wierenga et al., 2014) and are highly interconnected with the PFC, such that it has been suggested their maturation occurs in concert (Bessières et al., 2019). One study has shown that neuronal projections from the PFC to the basolateral-amygdala are pruned in late adolescence around PND 45 (Cressman et al., 2010) though the involvement of microglia or complement was not examined. Interestingly, C1q and iC3b/CR3 and microglia mediated synaptic pruning has recently been observed in the rat central nucleus of the amygdala (CeA) (Yuan et al., 2021). Here, microglial synaptic engulfment was exacerbated by chronic corticosterone exposure (Yuan et al., 2021) and ameliorated by environmental enrichment (Yuan et al., 2021). Whether these mechanisms are also at play under physiological conditions during amygdala maturation will be of interest.

In summary, while there is some evidence for a role of complement in adolescent synaptic pruning, our understanding is far from complete. Many studies of complement dependent pruning in brain regions beyond the visual system have focused on earlier windows of development, or the adolescent period has been omitted and comparisons made between early postnatal and adult timepoints only. In addition, while several studies demonstrate that microglial-dependent synaptic pruning does indeed occur during adolescence, these studies have not always investigated whether complement is involved. Detailed timepoint studies are therefore needed to clarify the involvement of complement in adolescent synaptic pruning of key brain areas linked to psychiatric disease with an onset during this stage of life.

The available data does however demonstrate that complement mediated synaptic pruning is extremely nuanced not just in terms of developmental timing and region, but also in terms of synapse specificity. Previous studies have suggested that microglial phagocytosis targets presynaptic material (Paolicelli et al., 2011; Schafer et al., 2012) and postsynaptic synapses are excluded or pruned *via* alternative, unknown mechanisms. Furthermore, inhibitory synapses seem to be less vulnerable to complement-mediated synaptic engulfment (Zhan et al., 2014; Shi et al., 2015; Salter et al., 2020). Concurrently, certain types of dendritic spine may be more or less susceptible to synaptic pruning, with reports of selective loss of filopodia and medium-sized spines in complement driven excessive pruning (Comer et al., 2020).

## What Are the Long-Term Impacts of Aberrant Complement-Mediated Synaptic Pruning?

The consequences of dysfunctional developmental synapse elimination for adult brain structure and function are only just beginning to be understood. Regarding synaptic pruning deficits resulting from complement and microglial disruption, it is not clear whether such changes represent a reduction in pruning occurring at the correct time, or a temporal shift in normal pruning, both of which have potential to cause long-term deficits in functional connectivity. In one study, a transient delay in pruning caused by TREM2 deficiency was sufficient to cause persistent electrophysiological and pharmacological phenotypes of the hippocampal CA1 area consistent with a failure of normal maturation processes (Paolicelli et al., 2011).

In addition, there have been few neuroimaging studies of animal models of abnormal synaptic pruning and thus the long-term macrostructural impacts of abnormal developmental synaptic pruning are not well characterised, although decreased synaptic pruning has been associated with reduced functional connectivity in resting-state fMRI (Zhan et al., 2014). Another study reported that deficient synaptic pruning resulted in functional underconnectivity of hippocampal networks in the absence of structural changes in white matter (Filipello et al., 2018). To the best of our knowledge there have been no longitudinal imaging investigations of animal models displaying abnormal developmental synaptic pruning. Such studies could add to our knowledge by capturing the evolving functional and structural connectivity patterns resulting from abnormal developmental pruning. Moreover, they would be of value in revealing any early functional readouts of abnormal synaptic pruning occurring at adolescence or in earlier developmental windows that could have translational value as biomarkers in diseases associated with abnormal synaptic pruning.

A range of behavioural phenotypes have been reported in mouse models displaying aberrant synapse elimination (summarised in Table 2). Intriguingly, excessive PFC synaptic pruning due to *C4A* overexpression is associated with changes in behavioural domains that closely mirror the negative symptomatology of schizophrenia. Mice overexpressing *C4A* displayed elevated anxiety, altered social interactions, spatial working memory deficits and repetitive behaviours, although pre-pulse inhibition and depressive-like behaviours were unaffected (Comer et al., 2020; Yilmaz et al., 2021). Several other studies have reported behavioural phenotypes in complement knockout models and other manipulations (see Table 2). C3aR deficient mice show marked elevated anxiety-like behaviour and stress responses to anxiety-provoking situations (Pozo-Rodrigálvarez et al., 2021; Westacott et al., 2022) as well as alterations in hippocampal, amygdala and cortical volume (Pozo-Rodrigálvarez et al., 2021). Anxiety phenotypes have also been reported in mice exposed to excessive pre-natal complement activity (Girardi et al., 2015). In contrast, when C3 is constitutively knocked out, animals show subtle signs of reduced anxiety at 3–6 months of age (Westacott et al., 2022) but this phenotype is more pronounced in aged (16-month-old) mice (Shi et al., 2015). Moreover, C3 but not C3aR deficient mice show heightened responses in fear conditioning paradigms (Wang et al., 2020; Westacott et al., 2022) and improved hippocampal spatial memory (Perez-Alcazar et al., 2013; Shi et al., 2015). Whether these phenotypes are specifically linked to disruptions in developmental complement-mediated synaptic pruning has not been investigated. However, several studies now highlight associations between perturbed pruning and anxiety, suggesting that brain circuits associated with emotion might be particularly vulnerable.

**TABLE 2:** Studies reporting manipulations of complement or microglial pathways implicated in developmental synaptic pruning.

Study (references)	Model	Phenotypes (synaptic, electrophysiological, behavioural or functional connectivity)
Zhan et al., 2014	*Cx3cr1* knockout mouse	Impaired synaptic pruning Weak synaptic transmission Decreased functional connectivity Deficits in social interaction Repetitive behaviours
Filipello et al., 2018	*TREM2* knockout mouse	Impaired synaptic pruning Enhanced excitatory neurotransmission Decreased functional connectivity Altered social behaviour Repetitive behaviours
Yilmaz et al., 2021	Mouse model of human *C4A* overexpression	Excessive synapse pruning and reduced cortical synapse density Altered social behaviour Elevated anxiety-like behaviour Working memory deficits
Comer et al., 2020	Overexpression of mouse *C4* in mPFC	Enhanced microglial engulfment of synaptic material and dendritic spine deficits Decreased synaptic connectivity Altered social behaviour Repetitive behaviour
Westacott et al., 2021	*C3* knockout mouse, *C3aR* knockout mouse	Increased adult hippocampal neurogenesis in *C3*^–/–^ mice Impaired reversal learning/perseveration in in *C3*^–/–^ mice
Westacott et al., 2022	*C3* knockout mouse, *C3aR* knockout mouse	Elevated anxiety-like behaviour and stress responses in *C3aR*^–/–^ mice Increased fear reactivity/fear memory recall in *C3*^–/–^ mice
Pozo-Rodrigálvarez et al., 2021	*C3aR* knockout mouse	Altered morphology of amygdala, hippocampus, somatosensory and motor cortices Hyperactivity Elevated anxiety-like behaviour
Shi et al., 2015	*C3* knockout mouse	Protection from age-dependent hippocampal synapse loss and plasticity Enhanced spatial and fear memory in aged mice Reduced anxiety-like behaviour in aged mice
Perez-Alcazar et al., 2013	*C3* knockout mouse	Increased number of hippocampal CA3-CA1 excitatory synapses Reduced presynaptic glutamate release probability Enhanced spatial & reversal learning
Wang et al., 2020	Complement inhibition in contextual fear memory acquisition and forgetting *via* CD55 (cre-dependent AAV expressing CD55/DAF injected into mouse dentate gyrus)	Better fear memory recall Higher reactivation rate of engram cells Fewer microglia containing synaptic material from engram cells
Girardi et al., 2015	Mouse model of pregnancy complications harbouring placental C3 deposition and foetal brain C3 deposition	Disrupted offspring cortical axonal cytoarchitecture Elevated anxiety-like behaviour in offspring

## Clarifying the Nature of Microglia–Synapse Interactions

Precisely how microglia interact with synapses during complement-mediated elimination remains a topic of debate. Due to practical challenges surrounding the observation of microglial-synapse interactions *in vivo*, evidence so far has been indirect, e.g., observations of increased synapse density in *ex vivo* fixed tissue from models of genetically engineered complement disruption. While such results are convincing due to having been observed across multiple models, such data can reveal little about the physical actions of microglia at the synapse. Another indirect source of evidence comes from the presence of synaptic material within microglial lysosomes observed *via* confocal, electron and super-resolution microscopy (Stevens et al., 2007; Paolicelli et al., 2011; Schafer et al., 2012, 2014; Weinhard et al., 2018). A caveat of these data is that it is possible that synaptic material found within microglia is a consequence of microglial uptake and clearance of debris from apoptotic neurons, which are commonplace during development (Nijhawan et al., 2000). Therefore, whether microglia actively prune synapses or merely ‘clean up’ is uncertain.

Recently, more direct evidence has come from the mapping of dynamic microglia–synapse interactions using correlative light and electron microscopy of hippocampal slice cultures. A fascinating study by Weinhard et al. (2018) demonstrated that microglia do directly and actively engulf and eliminate synaptic material. However, no evidence was found for complete phagocytosis, and instead microglia were observed to engage in ‘trogocytosis’ or ‘nibbling’ of presynaptic structures. This form of partial phagocytosis observed in hippocampal slice cultures was also independent of complement CR3 signalling. However, a subsequent study by Lim and Ruthazer (2021) found that C3 did significantly influence the active microglial trogocytosis of retinal ganglion cell axons in the developing *Xenopus laevis* retinotectal circuit. In relation to the discrepancy between these results and the aforementioned study, the authors suggest that compensation may occur in CR3 knockout mice through the multiple other C3 receptors (Lim and Ruthazer, 2021).

## Contribution of Other Glial Cells to Synaptic Elimination

Microglia are unlikely to be complement’s only sidekick in synaptic pruning. Instead, there is likely a complex interplay of neuronal and glial cells. Astrocytes share with microglia the capacity to actively engulf synapses in an activity-dependent manner during development, but so far astrocytic-dependent elimination appears to be complement independent and instead rely on molecules such as MEGF10 and MERTK (Chung et al., 2015). Mice deficient in these proteins show highly similar dLGN phenotypes to C1q, C3 and C4 knockout mice, characterised by an abundance of functional synapses remaining beyond the critical window of refinement, suggesting redundancy exists in mechanisms for visual system pruning. In addition, cytokines released by astrocytes such as IL-33 promotes microglial-synapse engulfment under physiological conditions (Vainchtein et al., 2018). A previous report describing interactions between C1q and transforming growth factor-β (TGF-β) secreted by astrocytes during the critical period of development has recently been withdrawn (Bialas and Stevens, 2013) and thus the precise role of astrocytes in complement mediated pruning remains unclear. It has been reported that C3a secreted by astrocytes interacts with neuronal C3aR to regulate dendritic spine density and synaptic plasticity in culture (Lian et al., 2015). Since astrocytes are a key source of complement proteins and receptors (Sayah et al., 2003; Stephan et al., 2012; Lian et al., 2015) it is likely that astrocytes do play important initiating and supporting roles in complement and microglial mediated synaptic elimination, but further research is needed.

In addition, an intriguing role for oligodendrocyte precursor cells (OPCs) has been described in a recent preprint by Buchanan et al. (2021). The authors used large scale serial electron microscopy to examine distinct glial cell types in the mouse visual cortex and reported that much like microglia, the processes of OPCs often contacted terminal axon branches and possessed abundant phagolysosomes containing presynaptic material. Strikingly, during adolescence these phagolysosomes were observed significantly more in OPCs than in microglia, suggesting that this underappreciated cell type may play a substantial role in the refinement of neuronal circuits at this later stage of development.

## What Signals Determine Which Synapses Get Removed?

One of the most significant outstanding questions is how complement activation leads to the selective removal of synapses. It is still unclear whether C1q/C3 tag all synapses, or just a subset of weak or inactive synapses, and the precise mechanisms by which ‘to be pruned’ synapses are demarcated from others. One candidate mechanism involves apoptotic-like processes localised within the synaptic compartment, which can trigger C1q-associated synaptic pruning (Györffy et al., 2018). Interestingly, the transient externalisation of the normally cytoplasm-facing phospholipid phosphatidylserine (PS) in a subset of synapses on non-apoptotic neurons during development may constitute a ‘eat-me’ signal for microglia and presynaptic colocalisation of C1q and PS is developmentally regulated in the dLGN (Scott-Hewitt et al., 2020). It is also possible that complement regulatory molecules could protect ‘strong’ synapses from tagging or engulfment. In support of this notion, although expression of many of the known complement inhibitors has not previously been detected in neurons (Cahoy et al., 2008), Cong et al. (2020) reported that a neuronally expressed complement inhibitor SRPX2 binds directly to C1q to block its activity. Knockdown of this protein led to elevated C3 deposition and microglial synapse engulfment (Cong et al., 2020).

## Do Other Complement Pathways Also Participate in Synaptic Pruning?

Since the first report of complement-mediated synaptic pruning in 2007, research has continued to focus on the C3b/CR3 axis (Stevens et al., 2007; Schafer et al., 2014; Sekar et al., 2016) while other branches of the complement system have remained relatively unexplored. In particular, C3aR is a neighbouring pathway equally as important in its immune properties; it recruits phagocytic cells to sites of injury or infection (Coulthard and Woodruff, 2015) and is highly expressed on microglia (Quell et al., 2017), making it well placed to mediate a process such as synapse engulfment. In support of this, the C3a/C3aR axis drives synaptic elimination by microglia in neurodegenerative conditions such as tau-pathology (Litvinchuk et al., 2018) and West Nile virus infection (Vasek et al., 2016), and can modify microglial reactivity in the ageing brain (Propson et al., 2021) but whether this pathway plays a role in synaptic pruning in the developing brain is unverified. Other anaphylatoxins produced through complement activation, such as C5a and C4a also possess potent chemotactic properties and can recruit macrophages to the site of injury or inflammation (Klos et al., 2009; Zhou, 2012) making them well poised to participate in synaptic engulfment. In addition, receptors such as the complement receptor 4 (CR4, also known as cd11c) which is expressed on microglia and shares structural homology with CR3 as well as overlapping ligand specificity for iC3b, can induce phagocytosis by peripheral macrophages (Lukácsi et al., 2017). To our knowledge, this receptor has not been explored in the context of synaptic elimination in the CNS but could contribute to possible compensation in CR3 deficient mice. Finally, elements of the terminal complement pathway beyond C5b have not been investigated, although one may speculate that these may be capable of removal of synaptic structures for example *via* MAC formation (Cole and Morgan, 2003).

## Does Complement Tag Other Substrates for Removal During Development?

In a fascinating study by Kopec et al. (2018), C3/CR3 signalling was shown to mediate the targeted microglial removal of dopamine D1 receptors (D1rs) in the rodent nucleus accumbens during adolescence. While adolescent downregulation of D1rs is seen in both sexes, the mechanisms were found to be CR3 dependent in males only. Furthermore, changes in D1r expression patterns paralleled the emergence of social play behaviours in males, suggesting causal links between complement-mediated receptor removal and developmental behavioural change. Microglial engulfment of various substrates is a critical aspect of development, such as the removal of excess cortical neuronal precursor cells (Cunningham et al., 2013) and oligodendrocyte precursors in the corpus callosum (Nemes-Baran et al., 2020). Whether complement plays a role in these other forms of developmental refinement is unknown. Microglia also induce apoptosis and engulf adult born stem cells in the dentate gyrus neurogenic niche (Sierra et al., 2010). Our own data has shown that deficient C3 signalling leads to greater survival of adult born hippocampal stem cells (Westacott et al., 2021) but whether this phenotype is related to potentially altered microglial-apoptosis coupled phagocytosis has not been investigated.

## Complement, Microglia and Extracellular Matrix Interactions

Potential interactions between complement and microglia with the extracellular matrix (ECM) in synaptic remodelling may be of relevance given the growing evidence of ECM abnormalities in psychiatric disorders (reviewed in Pantazopoulos and Berretta, 2015). The ECM is a highly organised molecular mesh that surrounds cells and consists of glycoproteins, proteoglycans and collagens amongst other proteins (Novak and Kaye, 2000). Within the ECM, perineuronal nets (PNNs) are condensed ECM assemblies that encapsulate the soma and proximal dendrites of neurons. The ECM is now regarded as a key component of synaptic organisation that facilitates function and regulates the contact between glia and synapses (Fawcett et al., 2019). In schizophrenia, PNN density is reduced in the PFC (Mauney et al., 2013) mirroring the commonly observed synaptic loss in this area, whereas genetic and histological data implicate perturbation of ECM signalling pathways in ASD (Pantazopoulos and Berretta, 2015). The mechanisms underlying these observations may relate to the developmental roles of ECM components. Beyond stabilising the synaptic architecture, the ECM supports vital cellular functions including plasticity (Fawcett et al., 2019). PNNs are highly sensitive to modulation by environmental stimuli, and their formation and maturation temporally parallels the end of critical periods of plasticity (Pizzorusso et al., 2002). Mice lacking key PNN components show extended critical periods for ocular dominance plasticity (Bruckner et al., 2000) suggesting that PPNs are important for closing periods of heightened plasticity, and reactivation of plasticity is associated with degradation of PNNs (Murase et al., 2017). This is consistent with observations that the density of PNNs progressively increases throughout the peripubertal and adolescent period of synaptic refinement in the human PFC (Mauney et al., 2013).

Evidence for interactions between the immune system and ECM during developmental synaptic refinement is beginning to emerge. Recent work suggests that microglia may sculpt not only synapses, but also the ECM in which they are embedded. Nguyen et al. (2020) demonstrated that neuronal IL-33 promotes microglial engulfment of the ECM proteoglycan aggrecan in an activity-dependent manner, suggesting that microglia may locally clear the ECM around individual synapses during synaptic remodelling (Nguyen et al., 2020). In addition, PNNs are more abundant in the brain after microglia depletion, further supporting a role of microglia in dismantling PNNs (Crasper et al., 2020). There is potential for complement to insect with these processes, but further research is needed. In the periphery, several constituents of the ECM can directly bind and activate C1q, the alternative pathway and complement regulators (Groeneveld et al., 2005; Sjoberg et al., 2009; Fernandez-Godino et al., 2018; Gialeli et al., 2018). However, to the best of our knowledge, interactions between complement, ECM and microglia is yet to be investigated in the CNS but is a highly promising avenue of future research.

## Other Developmental Roles of Complement and Their Relation to Synaptic Pruning

Emerging evidence implicates physiological roles of complement in multiple aspects of neurodevelopment (reviewed in Coulthard et al., 2018a) including embryonic neurogenesis (Hawksworth et al., 2014; Coulthard et al., 2017, 2018b), neuronal precursor migration (Gorelik et al., 2017), neurite extension and neuronal morphology (Shinjyo et al., 2009; Moriyama et al., 2011; Lian et al., 2015), regulation of several stages of adult neurogenesis (Rahpeymai et al., 2006; Moriyama et al., 2011; Westacott et al., 2021), synaptogenesis (Stokowska et al., 2017) and learning and memory (Perez-Alcazar et al., 2013; Shi et al., 2015; Wang et al., 2020; Westacott et al., 2021). These processes likely have the potential to influence synaptic strength, plasticity and thus pruning. For example, the changes in synapse density seen in complement knockout models could also be related to altered synaptogenesis. Since many studies of complement-dependent synaptic pruning have utilised constitutive knockouts in which synaptic pruning may be influenced by altered connectivity in afferent brain regions, constitutive and cell-specific knockouts will be required to gain more refined information about the influence of complement on developmental synaptic pruning.

## Therapeutic Potential for Targeting Complement-Mediated Synaptic Pruning

Given the mounting evidence surrounding the role of complement in the pathophysiology of disorders such as schizophrenia, therapies aimed at modulating the synaptic pruning process are of obvious interest. The second-generation tetracycline antibiotic minocycline is known to inhibit microglia (Kobayashi et al., 2019), has high brain penetrance and is well tolerated in humans, making it an obvious first port of call. Furthermore, *in vitro* studies have shown minocycline to be effective in decreasing microglial synapse uptake in a dose dependent manner (Sellgren et al., 2019). There have already been human trials of minocycline as an adjunctive treatment in individuals with active schizophrenia-spectrum disorders, but so far results have been mixed. Two meta-analyses of randomised control trials found that adjunctive minocycline treatment improved multiple symptom dimensions (Solmi et al., 2017; Xiang et al., 2017), but two subsequent large trials reported no clinical benefits over and above routine clinical care (Deakin et al., 2018; Weiser et al., 2019). However, it is likely that by the time symptoms manifest, neurodevelopment has already been significantly derailed in the years prior. Studies of individuals at clinically high risk for psychosis have found reductions in cortical grey matter thickness preceding onset of symptoms (Cannon et al., 2015). Thus, earlier interventions may be warranted, potentially targeting the period of peak synaptic pruning in the most affected brain areas. In a retrospective analysis of electronic health records, there was a 40% reduction in psychosis incidence in adults who received minocycline treatment for acne as adolescents, compared to other classes of antibiotics (Sellgren et al., 2019) suggesting that targeting synaptic pruning during the period of cortical maturation may be a more promising approach. Identifying who to treat and when, as well as the ethical issues surrounding treatment of children who may never go on to develop overt psychopathology, require careful consideration. Identification of prodromal states or clinical high risk or ‘ultra-high risk’ individuals may provide a window of opportunity for intervention, but further studies are needed to understand the likely relationship of prodromal presentation to underlying synaptic and inflammatory changes.

While microglia-targeted therapies certainly hold potential, another avenue of investigation is whether complement activation could be targeted to prevent hyperactive synaptic elimination. Several complement targeted therapeutics exist for neurological conditions (reviewed in Mastellos et al., 2019) with potential to be repurposed for psychosis and other disorders such Alzheimer’s disease. However, as with any complement targeted therapies, nuanced approach would be needed to avoid unwanted disruption of necessary immune actions. Interfering with C3, or disrupting opsonins that tag synapses, such as C1q or C3b, would likely create significant vulnerability to bacterial or viral infections. In addition, the possibility of disturbing ongoing homeostatic functions of complement in the adult brain such as regulating adult neurogenesis and synaptic plasticity (Wang et al., 2020; Westacott et al., 2021) have to be considered. However, if as recent studies suggest, complement can contribute but is not necessary for synaptic pruning in the healthy brain (Sager et al., 2021; Yilmaz et al., 2021), then targeted therapies to reduce high C4A levels in carriers of risk-associated variants (Sekar et al., 2016) might be effective without disrupting the normal underlying synaptic pruning processes.

Further studies utilising a multi-modal approach in humans (featuring ultra-high-risk groups or early psychosis) and animal models are needed to advance the field. At present, we have little understanding of both the normal synaptic pruning processes taking place in the adolescent brain, and how they are perturbed in neurodevelopmental disorders and this is a significant barrier in knowing when interventions should be targeted. Prospective longitudinal studies in clinical-high risk groups stratified by C4A status utilising recent developments in neuroimaging techniques would be of great value to the field. For example, SV2A positron emission tomography (PET) has been used to demonstrate reduced synaptic density in patients with schizophrenia (Onwordi et al., 2020), depression (Holmes et al., 2019) and Alzheimer’s disease (Mecca et al., 2020) and can readily be applied in preclinical models. In addition, neurite orientation dispersion and density imaging (NODDI) modelling of diffusion MRI data has proven sensitive to developmental changes in neurite density (Genc et al., 2017) and the effects of inflammation on brain microstructure (Dowell et al., 2019).

## Conclusion

Synaptic pruning is an important aspect of neurodevelopment that is increasingly implicated in risk for psychiatric disorders. Interacting with microglia in particular, the complement system plays a key role in mediating this process especially in early postnatal critical periods such as those extensively characterised in the visual system. The evidence for complement mediated pruning in later sensitive windows of development such as adolescence, which is associated with enhanced susceptibility to a range of psychiatric disorders, is less clear. The few studies available at present suggest that while complement may not be involved in adolescent synaptic pruning of regions such as the PFC under physiological conditions, animal work does support the notion that disruption of normal adolescent synaptic pruning processes are a key pathophysiological mechanism in schizophrenia. It is possible that similar mechanisms are at play in other neurodevelopmental disorders associated with altered synaptic density or cortical volume changes. Whether synaptic pruning is a general mechanism throughout the brain, or whether selective neurons, circuits and synapses are eliminated and when remains mainly unknown and due to the heterogeneity in developmental trajectories, this is a major challenge for this area of research. Detailed preclinical studies including measures of complement-mediated synaptic pruning in yet unexplored brain regions, captured at prepubertal, adolescent and adult timepoints will be of importance in providing fundamental insights into how circuits develop to influence adult brain structure, function and behaviour in both the healthy brain and in psychiatric conditions and also in highlighting potential therapeutic targets, populations and time points for effective interventions.

## Author Contributions

Both authors listed have made a substantial, direct, and intellectual contribution to the work, and approved it for publication.

## Conflict of Interest

The authors declare that the research was conducted in the absence of any commercial or financial relationships that could be construed as a potential conflict of interest.

## Publisher’s Note

All claims expressed in this article are solely those of the authors and do not necessarily represent those of their affiliated organizations, or those of the publisher, the editors and the reviewers. Any product that may be evaluated in this article, or claim that may be made by its manufacturer, is not guaranteed or endorsed by the publisher.
